# Coated Flow Diverters and Single Antiplatelet Treatment: Where are We?

**DOI:** 10.1007/s00270-022-03185-0

**Published:** 2022-07-11

**Authors:** Laurent Pierot

**Affiliations:** Department of Neuroradiology, CHU Reims, University Reims Champagne Ardenne, 45 rue Cognacq-Jay, 51100 Reims, France

We have read with a great interest the impressive paper of Hellstern et al. [1]. It is the first large series (102 patients with 132 aneurysms) reporting the use of a coated flow diverter (p64-MW-HPC; phenox, Bochum, Germany) under prasugrel single antiplatelet treatment (SAPT) analyzing safety and short-term efficacy. While the rates of periprocedural and postprocedural complications are relatively high (13.6% and 6.8%, respectively), most periprocedural complications were technical without clinical worsening and no periprocedural thromboembolic or hemorrhagic complication was observed. Postprocedural and delayed complications occurred in 9/102 patients (8.8%) with clinical deterioration in 4/102 (3.9%). This great safety of aneurysm treatment with a coated flow diverter and SAPT is associated with good short- and mid-term efficacy. At short-term follow-up (70–180 days), complete aneurysm occlusion was observed in 64/95 aneurysms (67.4%) and in 58/74 aneurysms (78.4%) at mid-term follow-up (181–500 days). These results suggest that the strategy p64-MW-HPC + SAPT results potentially in improved occlusion rates.Kindly check and confirm ON is correctly identified for Affiliation 1.Correct.Kindly check and confirm Conflict of interest statement is correctly identified.It is ok.

Due to its high efficacy, flow diversion is increasingly used for the endovascular treatment of intracranial aneurysms [2]. If its indication was initially restricted to large and giant internal carotid artery (ICA) unruptured aneurysms and recanalized aneurysms, there was a progressive expansion to more distal and bifurcation aneurysms. However, due to the need for dual antiplatelet treatment (DAPT) before and after the treatment with a flow diverter, the use of this technique is still restricted to unruptured and recanalized aneurysms. To overcome this limitation coated flow diverters have been developed. In fact, different surface modifications (“coatings”) are currently under evaluation with different goals: improving navigability of the flow diverter, accelerating the endothelialization of the flow diverter, or reducing platelet aggregation on the flow diverter.

Hydrophilic Polymer Coating (HPC; phenox GmbH, Bochum, Germany) is made from a glycocalyx-like glycan-based polymer covalently bonded to the surface of flow diverters or other devices. In vitro experiments and animal studies have shown that HPC reduces platelet aggregation on the p64-MW-HPC flow diverter [3]. The HPC surface modification aims to reduce platelet aggregation on the flow diverter, reduce thromboembolic complications, and minimize APT pre-and post-procedure. The large series of Hellstern et al. is the first to report the use of a coated flow diverter under prasugrel SAPT. It shows the very high safety of this strategy with no periprocedural thromboembolic complications and limited postprocedural complications. This series is an important milestone in the evaluation of the coated flow diverter p64-MW-HPC. As suggested by the authors, the next step will be to compare treatment of intracranial aneurysms with a bare flow diverter under DAPT (which is the current clinical practice) to the treatment with p64-MW-HPC under SAPT. COATING (Coating to Optimize Aneurysm Treatment in the New Flow Diverter Generation) is a randomized controlled trial (RCT), which was designed to compare the safety and efficacy of aneurysm treatment in patients treated with bare p64-MW under DAPT and patients treated with coated p64-MW-HPC under SAPT [4]. This multicenter, European study is currently including patients and is the first to evaluate in a comparative way a coated flow diverter under SAPT.

To conclude, due to its high efficacy, flow diversion is increasingly used in the endovascular management of intracranial aneurysms. Due to the need of pre and postoperative DAPT, the use of flow diversion is still restricted to unruptured and recanalized aneurysms. For the first time, a large single-arm study is showing the high safety of aneurysm treatment with a coated flow diverter under SAPT. The next step is now to compare the treatment with a bare flow diverter under DAPT and a coated flow diverter (p64-MW-HPC) under SAPT (COATING study underway).
